# Detecting at-risk mental states for psychosis (ARMS) using machine learning ensembles and facial features

**DOI:** 10.1016/j.schres.2023.07.011

**Published:** 2023-08

**Authors:** Alexandre Andrade Loch, João Medrado Gondim, Felipe Coelho Argolo, Ana Caroline Lopes-Rocha, Julio Cesar Andrade, Martinus Theodorus van de Bilt, Leonardo Peroni de Jesus, Natalia Mansur Haddad, Guillermo A. Cecchi, Natalia Bezerra Mota, Wagner Farid Gattaz, Cheryl Mary Corcoran, Anderson Ara

**Affiliations:** a Laboratório de Neurociencias (LIM 27), Instituto de Psiquiatria, Hospital das Clínicas HCFMUSP, Faculdade de Medicina, Universidade de Sao Paulo, Sao Paulo, SP, Brazil; b Instituto Nacional de Biomarcadores em Neuropsiquiatria (INBION), Conselho Nacional de Desenvolvimento Científico e Tecnológico, Brazil; c Instituto de Computação, Universidade Federal da Bahia, Salvador, BA, Brazil; d IBM T.J. Watson Research Center, Yorktown Heights, NY, USA; e Instituto de Psiquiatria (IPUB), Departamento de Psiquiatria e Medicina Legal, Universidade Federal do Rio de Janeiro (UFRJ), Rio de Janeiro, Brazil; f Research Department at Motrix Lab – Motrix, Rio de Janeiro, Brazil; g Icahn School of Medicine at Mount Sinai, New York, NY, USA; h Statistics Department, Federal University of Paraná, Curitiba, PR, Brazil; i James J. Peters VA Medical Center Bronx, NY, USA

**Keywords:** Schizophrenia, Clinical high risk, Random forest, Avolition, Emotion, Hallucinations, Delusions

## Abstract

**Aims:**

Our study aimed to develop a machine learning ensemble to distinguish “at-risk mental states for psychosis” (ARMS) subjects from control individuals from the general population based on facial data extracted from video-recordings.

**Methods:**

58 non-help-seeking medication-naïve ARMS and 70 healthy subjects were screened from a general population sample. At-risk status was assessed with the Structured Interview for Prodromal Syndromes (SIPS), and “Subject's Overview” section was filmed (5–10 min). Several features were extracted, e.g., eye and mouth aspect ratio, Euler angles, coordinates from 51 facial landmarks. This elicited 649 facial features, which were further selected using Gradient Boosting Machines (AdaBoost combined with Random Forests). Data was split in 70/30 for training, and Monte Carlo cross validation was used.

**Results:**

Final model reached 83 % of mean F1-score, and balanced accuracy of 85 %. Mean area under the curve for the receiver operator curve classifier was 93 %. Convergent validity testing showed that two features included in the model were significantly correlated with Avolition (SIPS N2 item) and expression of emotion (SIPS N3 item).

**Conclusion:**

Our model capitalized on short video-recordings from individuals recruited from the general population, effectively distinguishing between ARMS and controls. Results are encouraging for large-screening purposes in low-resource settings.

## 1 Introduction

Schizophrenia is the most burdensome disease among neurological and psychiatric disorders, encompassing severe disability in early life (Whiteford et al., 2015). To counter that, the at-risk mental state (ARMS)—or the clinical/ultra-high risk (CHR or UHR) for psychosis—was conceptualized nearly three decades ago to prevent schizophrenia spectrum disorders (Fusar-Poli et al., 2013). Despite being one of the most studied preventive paradigms in psychiatry, concern still remains about its clinical use, namely accuracy and practical applicability (Woodberry et al., 2021). As for accuracy, ARMS criteria has a predictive value of psychosis onset within the ensuing two years of less than one third of cases (Kempton et al., 2015), relevant in respect to false-positives and stigma (Colizzi et al., 2020). Regarding practical applicability, for ARMS—and for psychiatry in general—there is an absence of objective clinical tests of the type routinely used in other fields of medicine (Bedi et al., 2015). Currently ARMS diagnosis is made by using long interviews, like the Structured Interview for Prodromal Syndromes (SIPS) (McGlashan et al., 2001a) or the Comprehensive Assessment of At Risk Mental States (CAARMS) (Yung et al., 2002). They are time-consuming instruments that must be administered by trained and experienced interviewers in order to capture the nuances and subtleties of subclinical psychosis (Hinterbuchinger and Mossaheb, 2021). Self-report instruments are usually an alternative for screening and diagnosis in psychiatry, but not in the case of ARMS as there is both a statistically and practically significant difference when psychosis risk symptoms are assessed by self-report or by interview (Granö et al., 2016).

Given both of these difficulties, current trends in computational psychiatry point toward the viability of automated detection and characterization of ARMS individuals (Corcoran et al., 2018). They mainly focus on subtle, convenient and accessible information from subjects, such as that from verbal and non-verbal communication, for instance (Birnbaum et al., 2020). Such type of data could be acquired by filming the subject for a few minutes during specific tasks and submitting the footage to an automated artificial intelligence protocol, greatly shortening assessment time. In this sense, there is a significant number of studies addressing the analysis of verbal language features such as natural language processing (Corcoran et al., 2020), speech connectedness (Spencer et al., 2021), and acoustics (Stanislawski et al., 2021) in ARMS, eliciting encouraging results. However, verbal language may face the challenge of validity of findings between different idioms, and of reliability of transcriptions generated by automated processes.

Concerning non-verbal communication, though, comparatively fewer studies have been conducted up until now. Osborne and colleagues, for instance, observed a reduced frequency of rhythmic movements made with the hands during speech, which was related to elevated postural sway in 30 ARMS individuals compared to controls (Osborne et al., 2017). Kindler et al. have documented abnormal involuntary movements using the Abnormal Involuntary Movement scale in 45 ARMS subjects against 39 healthy controls. Scores were significantly higher in ARMS compared to controls, and they were correlated with regional blood flow differences in the prefrontal cortex and Brodmann area 6, and left middle frontal gyrus (Kindler et al., 2019). In a more comprehensive approach, Mittal's research group has shown motor slowing (Damme et al., 2020), more gestures made during pauses in speech (Millman et al., 2014), increased postural sway (Dean et al., 2015) and less gesticulation (Mittal et al., 2006) in ARMS individuals as compared to healthy individuals. Furthermore, they showed that movement abnormalities are correlated with symptom severity and may be predictive of psychosis onset (Mittal et al., 2007).

More recently, automated interfaces in which a software processes the images and generates metrics have been employed, such as the Motion Energy Analysis (MEA) (Lopes-Rocha et al., 2022). Dean et al. used the MEA to analyze 54 ARMS individuals and 62 healthy controls during the first 15 min of the SIPS interview (Dean et al., 2018). They found that ARMS individuals showed greater total body movement and speed of body movements, and lower variation in body movement compared to healthy controls (Dean et al., 2018). A recent study by Gupta et al. with 42 ARMS individuals compared to 42 matched controls showed that there were significant differences in the facial expressions of emotion between groups (Gupta et al., 2022). Using two automated facial analysis programs (iMotions and Facereader), authors adopted an ultrathin slicing approach—i.e., brief 1-minute videos—to show that these differences could be detected in small fragments of behavior.

At last, all these results were generated in help-seeking ARMS populations that were referred to specialized clinics—which are available in only a few countries—and population studies to address real-world validity of results and protocol applicability are lacking. As such, in this study we analyze 58 ARMS individuals and 70 healthy controls from the general population to build an automated classifier based on facial features extracted from these subjects' videos. The present study has some noteworthy methodological features in that (a) we examined ARMS and control individuals screened from an epidemiological sample, i.e., they were non-help-seeking and medication-naive; (b) we developed a robust machine-learning method to classify such individuals as ARMS or controls, using brief video data only (5–15 min), (c) we used facial landmarks in the machine-learning method instead of using pre-set combinations of landmarks, such as that encoded by facial action units. Our hypothesis is that an effective machine-learning algorithm can be built to detect ARMS status in individuals from the general population, using data derived from video images only.

## 2 Methods

### 2.1 Sample

This study is part of the Subclinical Symptoms and Prodromal Psychosis (SSAPP) Project, which consists of a population-based cohort study situated in São Paulo City, Brazil, involving over 2500 individuals aged 18–35 years (Loch et al., 2022b). First, individuals were interviewed by telephone using the Prodromal Questionnaire-Brief version (PQ-16) and the Basic Symptoms scale (BS), following previously published screening procedures (McDonald et al., 2019). The PQ-16 is a shorter version of the original 92 items used in the Prodromal Questionnaire (PQ) (Loewy et al., 2005), which consists of a self-report questionnaire with 16 items to screen for ARMS of developing psychosis (Ising et al., 2012). The BS is a criterion based on the basic symptoms of self-experienced disturbances in perception and cognition that are present in the initial manifestations of psychosis risk (Schultze-Lutter et al., 2010).

Then, individuals with a combined score > 10 on the PQ-16 + BS were called for a face-to-face interview at the Institute of Psychiatry, University of Sao Paulo, Brazil. They were assessed with the Structured Interview for Psychosis-Risk Syndromes (SIPS) (Diniz et al., 2021; McGlashan et al., 2001b) for ARMS status, and with the Structured Interview for DSM-5 diagnosis (SCID-5) (First et al., 2016). The SIPS is a structured diagnostic interview which diagnoses three prodromal syndromes for psychosis: the Brief Intermittent Psychotic Symptom syndrome (BIPS – experience of brief intermittent psychotic symptoms), the Genetic Risk and Deterioration syndrome (GRD – history of psychotic disorder in a first degree relative or schizotypal personality, and a decline of 30 % on the Global Assessment of Functioning Scale (GAF) in the past year) and the Attenuated Psychosis Syndrome (APS – presence of attenuated psychotic symptoms in the past year that are present at least once per week in the last month and have not reached a psychotic level) (Miller et al., 2003). The SCID-5 is a semi structured interview for the evaluation of DSM-5 disorders, including psychotic disorders. After these interviews, 58 individuals were determined as meeting criteria for ARMS and 70 as healthy comparison subjects.

Research was approved by the local and national ethics committee (National Committee on Research Ethics #1.709.439, University of Sao Paulo Ethics Committee #4.283.142).

### 2.2 Language protocols and data acquisition

Two protocols were applied, and audiovisual files collected by means of mobile phone positioned on a steady support, with participants sitting in front of the mobile phone. Mobile phones' native recording apps were used in the recordings (Android or IOs). Informed consent was provided by all participants, and approval by the Institutional Review Board at the University of Sao Paulo. The first protocol consisted of SIPS subject overview (SO), with the addition of an instruction to ask the subject to speak freely specifically about their childhood and relationship with their parents (Subject Overview—SO). This was an exploratory protocol, based on the lasting idea of troubled relationship with parents in psychoses described in the literature (Schiffman et al., 2002). The second was based on the paradigm of Mota (Mota et al., 2017, Mota et al., 2022), consisting of requesting oral memory reports (MR): a recent dream, an old dream and short-term memory reports based on 3 positively affective pictures—a baby, a puppy and a dessert. When participants did not remember a dream, they were prompted to describe the prior day. The rationale here—as well as in the first protocol—is to elicit the freest and most spontaneous speech possible, while also evoking an affectively meaningful discourse (Mota et al., 2017, Mota et al., 2022). After collection, video was immediately stored in a secure cloud service and deleted from the mobile. Protection was granted by means of current encryption protocols in the backend database and over the remote communications (SSL) according to Brazilian data protection compliance standards (Lei Geral de Proteção de Dados, LGPD; https://www.lgpdbrasil.com.br).

### 2.3 Ensemble machine learning methods

Three different Machine Learning algorithms were used. To reduce the number of features used in the final classifier, Gradient Boosting Machines were employed due to its ability to measure the number of times a feature is used to split data, therefore being useful to set an importance value to the features used. The classifier training was composed of a combination of AdaBoost with Random Forests as the weak learner, as such combination capitalizes on two ensemble methods to mitigate overfitting and improve error rates, as shown in other studies (Nayak et al., 2016; Putra et al., 2018; Thongkam et al., 2008). This section depicts each of them.

#### 2.3.1 Boosting methods

Boosting refers to a group of algorithms where weak classifiers (weak learners or base learners), classifiers that are slightly better than random guessing, are used in contribution, leading to a strong classifier capable of correctly predicting outcomes (Zhou, 2012). Sequentially, boosting algorithms modifies the training data according to the classifications performed in the previous step. The final prediction is than produced by a weighted contribution of each of the M weak learners as shown (Hastie et al., 2009):

$$G\left(x\right)=\mathrm{sign}\left(\Sigma_{m=1}^{M}\alpha_{m}G_{m}\left(x\right)\right).$$

where G_m_(x) is the weak classifier trained on step *m*, with weight *α*_m_ and *sign* being the signal function. The training data modification in step *m* is done as with the consequence of the predictions of step *m-1*: the observations that were incorrectly classified gain increased weight, whereas observations correctly classified have smaller weights attributed, in a way that, for every successive iteration, harder to classify samples receive higher influence (Hastie et al., 2009). In this work, Gradient Boosting Machines and AdaBoost were employed to achieve the final model.

#### 2.3.2 Gradient Boosting Machines

In Gradient Boosting Machines (Friedman, 2001, Friedman, 2002) (GBM), the boosting method is based on gradient descent, where the parameters of the weak classifiers are set seeking to minimize a loss function chosen prior to the beginning of the training (Natekin and Knoll, 2013). This minimization occurs by correlating the weak classifier at each iteration step with the negative gradient of the loss function (Natekin and Knoll, 2013).

Here GBMs were implemented with LightGBM (Ke et al., 2017). LightGBM is an implementation of GBM with Decision Trees as the base learner and two techniques to address challenges regarding the number of instances or features within the data used for training: Gradient-based One-Side Sampling (GOSS) and Exclusive Feature Bundling (EFB).

To reduce the amount of data in the training process, GOSS uses the gradient information to establish if a data instance is already well trained (small gradient) or not (large gradient), thus keeping the samples with large gradients and performing random sampling within instances with small gradients. The GOSS method helps the training algorithm to place particular attention on instances that did not perform well on the prior training step.

The second method, EFB, aims to reduce the number of features used during training. The algorithm finds mutually exclusive features and aggregates them into a small number of exclusive bundles in a greedy approximation implementation.

Due to the use of Decision Trees within its implementation, LightGBM has ways of analyzing the importance given to each feature in the dataset used for training: the number of times one feature was used to make a split in the Decision Trees used (“split” type) or the average information gain a feature provides when used to split a node in the Decision Trees (“gain” type).

#### 2.3.3 AdaBoost

The AdaBoost algorithm (Freund and Schapire, 1996) employs an additive weighted combination of weak learners and minimization of the exponential loss. Given a dataset *D*_*1*_, with samples *x*_*1*_, *x*_*2*_…*x*_*n*_, trained on base learners *G*_*1*_,*G*_*2*_…, we can generate other modified datasets (*D*_*2*_, *D*_*3*_…) that accentuate the misclassifications committed by each base learner. The first base learner *G*_*1*_ is trained with the original dataset, and its weight *α*_1_ is determined in a way that minimizes the loss function for the dataset employed in this step, than this weight is used in different ways create each new sample *x*_*i*_ of the dataset *D*_*2*_ that will be used in the next step: exp(−*α*1) if *x*_*i*_ was correctly classified or exp(*α*1) if *x*_*i*_ was misclassified (Zhou, 2012). This configuration makes the next iteration base learner to correct the mistakes of the previous one.

#### 2.3.4 Random Forest

Random Forests improve the Decision Trees algorithm by decorrelating the trees (James et al., 2021). More than one tree is built on bootstrapped samples (repeatedly pulling observations from the original dataset), but with a random number of features chosen as split candidates, instead of the full set. This configuration avoids the trees to use the stronger feature as the root node, which would result in similar, highly correlated, built trees that do not generalize well (James et al., 2021). The Random Forest with Decision Trees weakly correlated are more flexible, usually returning less test errors (Zhou, 2012).

### 2.4 Feature engineering and selection procedures

**Fig. 1:**
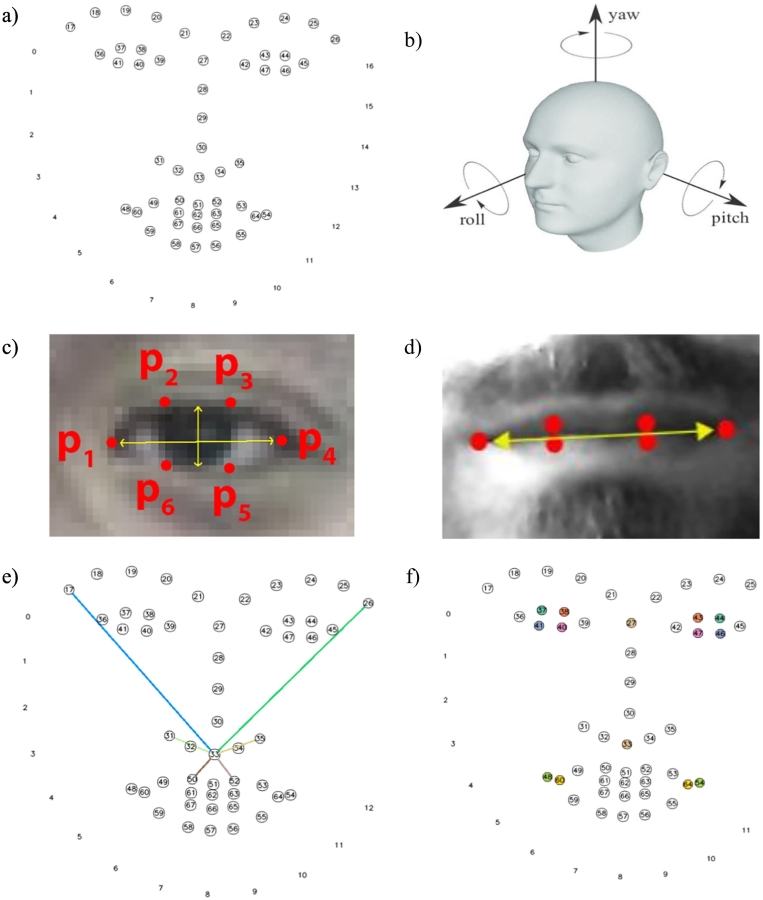
Facial landmarks used and feature interactions calculated. a) Facial landmarks extracted, circled points are the ones used in this work. b) Euler angles obtained from facial landmarks (Dubey and Tomar, 2022). c) Eye Aspect Ratio (EAR) (Soukupová and Cech, 2016). d) Mouth Aspect Ratio (MAR) (Bellino, 2018). e) Examples of interactions between point 33 and other face points. f) Matching pairs used to calculate Spearman's correlation coefficient.

In this work, since each video had different lengths, we used summary statistics instead of considering the time series related to the points in each video, thus being able to represent participants' movement throughout the entire interview. To summarize the data collected, Interquartile Range (IQR) and Median Absolute Deviation (MAD) were employed due to their robustness to outliers (Leys et al., 2013). In total, 649 features entered the machine learning model (Fig. 1). A detailed description of feature selection is available on Supplementary methods.

### 2.5 Training

Machine learning models used are a part of the scikit-learn (Pedregosa et al., 2011) library. We trained an AdaBoost algorithm with Random Forests as the weak learner. The AdaBoost algorithm was set with 900 Random Forest estimators, each having learning rate value set to 2, the Random Forests had 5 trees, with maximum depth of trees of 1 and the maximum features considered when splitting a tree was set to log2, other parameters were all the defaults present on the scikit-learn library.

To train and evaluate our model, we split data in 70/30 fashion: 70 % of available samples were used to train the algorithm and 30 % used to assess after fitting. As the samples used to create each splits might influence the model's final metrics, Leave-group-out (or Monte Carlo Cross Validation) was used, the method consists of splitting data in the chosen fashion multiple times, having different train and test groups created at each repetition and calculating metrics as the mean obtained in each test group evaluation (Kuhn and Johnson, 2013). Therefore, to avoid having influence on the train and test split chosen, we employed Monte Carlo Cross Validation, dividing 128 participants in 100 different 70/30 splits.

### 2.6 Metrics

To evaluate the results obtained, five different metrics were employed: Specificity, Sensitivity, F1 Score (harmonic mean between Precision and Sensitivity), Receiver Operating Characteristic Area Under the Curve (ROC-AUC) and Balanced Accuracy (arithmetic mean of the Sensitivity and the Specificity). For a detailed description of each of these metrics, please see Supplementary methods.

## 3 Results

### 3.1 General findings

The 58 participants meeting the ARMS criteria did not differ significantly to the 70 healthy controls in any demographic variable, but they did differ significantly in their SIPS scores (Table 1).

**Table 1:** Sample sociodemographic and clinical data.

	ARMS (*n* = 58)	Controls (*n* = 70)	p-Value
Age (mean, SD)	28.59 ± 4.44	28.31 ± 4.69	0.449^b^
Gender (male; n,%)	20 (34.48 %)	24 (34.29 %)	1^c^
Years of education (n; %) 0–9^a^	2 (0.03 %)	0	0.251^c^
10–12^a^	20 (34.48 %)	21 (30 %)	
13+^a^	35 (60.34 %)	49 (70 %)	
SIPS positive (mean, SD)	8.72 ± 3.02	3.80 ± 2.37	**<0.001**^b^
SIPS negative (mean, SD)	6.07 ± 4.47	4.36 ± 3.59	**0.031**^b^
SIPS disorganization (mean, SD)	2.71 ± 1.75	2.06 ± 1.66	**0.032**^b^
SIPS general (mean, SD)	5.88 ± 3.41	3.80 ± 3.10	**<0.001**^b^

**Bold**: significant associations.

a One of the ARMs participants did not have scholarly information annotated.

b Wilcoxon test.

c Fisher exact test.

**Fig. 2:**
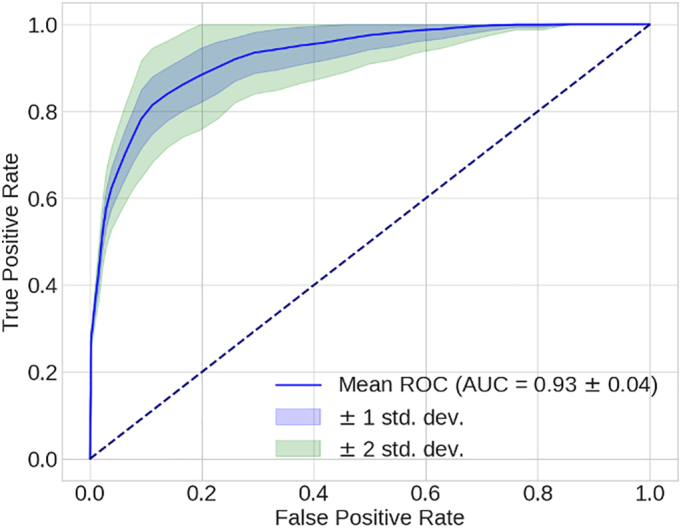
Average Receiver Operating Characteristic Curve and standard deviation.

To test a classification model, the experiments described here were made with the SO videos. Information of the MR videos are available as Supplementary materials (Tables 1S and 2S). We opted to show SO results because they performed slightly better. Model results are shown in Table 2. The True Positive and False Positive Rates are depicted in Fig. 2, showing the mean ROC Curve with its standard deviations.

**Table 2:** Machine learning model performance.

	Mean	Standard deviation	Maximum	Minimum
F1-score	83 %	±7 %	94.74 %	60 %
Specificity	89 %	±7 %	100 %	71.43 %
Sensitivity	81 %	±10 %	100 %	50 %
Balanced accuracy	85 %	±6 %	95.24 %	66.27 %
ROC-AUC	93 %	±4 %	98.94 %	78.84 %

### 3.2 AI model assembling

**Fig. 3:**
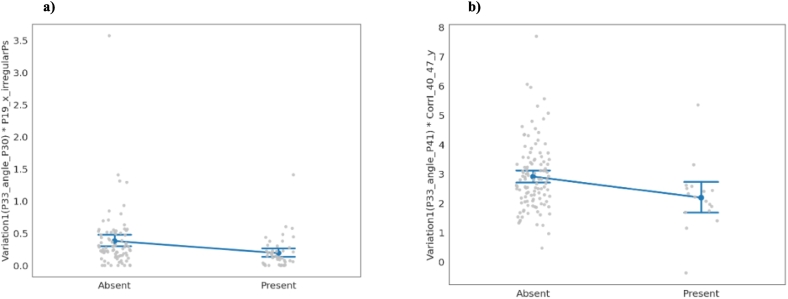
a) Representation of feature “face_33_angle_face30_mad ∗ face_19_x_outliers” (each point corresponds to one participant) and the estimative tendency of the mean with 95 % confidence interval (line joining categories) for the absence or presence from symptom N2 (Avolition). For this feature, the *p*-value of the Wilcoxon's Ranksum test is 0.0003. b) Representation of feature “face_33_angle_face41_mad ∗ spearman_40_47_y” (each point corresponds to one participant) and the estimative tendency of the mean with 95 % confidence interval (line joining categories) for the absence or presence of symptom N3 (Expression of Emotion). For this feature, the p-value of the Wilcoxon's Ranksum test is 0.011.

Concerning the model itself, after the steps of feature interaction and feature selection, most selected features are the ones obtained after repeated combination, representing the product of two different features. Only three features remained without any interaction: the Spearman's correlation coefficient between points 48 and 54, the partial autocorrelation coefficient of point 57 on the X axis and the percentage of the number of times the EAR was below the first quartile. All the other features had a role in the model. This was closely related to out-of-bootstrap validation (differs only by the number of training samples you draw) combinations of the original 649-dimension vectors. Therefore, some facial features and some of the Euler Angles calculated may appear more than once in the model. The counts of how many times each feature appears in the final model are presented in Fig. 1S (Supplement) — the counts for the MR videos are available in Fig. 3S.

### 3.3 Feature analysis

To inspect the features used in the model, Permutation Feature Importance was used, a technique to assess the importance of a given feature based on the score difference of the model after randomly changing it a number of times (Molnar, 2022). While training the AdaBoost algorithm with 100 different train and test splits, the features of the model were assessed on the test split using Permutation Feature Importance 100 times. The outputs of the changes in the F1 score after randomly changing one feature were summed and are presented in Fig. 2S (while the Permutation Feature Importance of MR videos are available in Fig. 4S in the Supplement). Only the 20 features with higher importance are shown; we added age, gender and scholar information even though their importance values are low.

### 3.4 Convergent validity

To better understand how the features describe differences between Control and ARMs groups (convergent validity), we depicted them with respect to the Negative symptoms found on SIPS. To do this, we separated individuals by the absence (SIPS score 0 or 1) or presence (score 2 or more) of each of the negative symptoms and used Wilcoxon's test to assess if individuals are drawn from the same distribution for each feature used on the final model. Fig. 3 shows two features for the negative symptom Avolition (N2) and Expression of Emotion (N3).

## 4 Discussion

Our study developed a machine learning (ML) algorithm that could effectively distinguish between individuals with the ARMS condition and healthy individuals from the general population with an AUC of 93 %, and a balanced accuracy of 84 %. This finding represents an important addition in the use of computer science behavioral analysis techniques to characterize human behavior in the context of mental health (Barron et al., 2022; Birnbaum et al., 2022).

To the best of our knowledge, our study is also the first to use facial movement to automatically detect ARMS individuals in a non-help-seeking sample derived from the general population. There are several studies addressing risk of conversion within ARMS samples—including the development of several risk calculators (Carrión et al., 2016; Lee et al., 2020; Oliver et al., 2021; Zhang et al., 2019)—but those aiming to distinguish between ARMS and control individuals are comparatively fewer. In an important cross-site validated study, which pooled 93 ARMS individuals from two different sites in the United States, the accuracy of a classifier to discriminate ARMS from first episode psychosis was of 72 % (Corcoran et al., 2018). Another classifier using metaphor-identification and sentiment analysis to automatically generate features among 34 ARMS individuals accurately identified 85 % of them against 17 first episode psychosis and 15 healthy control individuals (Gutiérrez et al., 2017). These types of studies are usually conducted in help-seeking populations, though. If we are striving for a convenient system that targets large-scale screening in psychosis, we need to aim for population samples, as emphasized above (Argolo et al., 2020).

As a strength of our protocol we highlight the use of an epidemiological sample constituted of non-help-seeking ARMS individuals from the general population. Evidence suggests that ARMS individuals frequently ask first for help from their family or friends (Fridgen et al., 2013), significantly delaying their sought for specialized mental health services—some estimate a duration of unrecognized risk for psychosis of 3 1/2 years (von Reventlow et al., 2014). Often multiple informal help-seeking pathways are attempted (Loch et al., 2019; Turner et al., 2006), and subjects usually reach out for help because of related affective symptoms and not because of the psychotic symptoms per se (Falkenberg et al., 2015). This delay is especially critical in settings where mental healthcare delivery is sub-optimal, such as in low and middle-income countries (Farooq et al., 2009)—also the case of the current study (Loch et al., 2016). As a result, studies from risk clinics might catch selected help-seeking individuals in a late pre-clinical stage (von Reventlow et al., 2014). Therefore, it is important to add naturalistic data from general population designs to the ARMS research knowledge. Especially if we understand that such results are closer to real-world scenarios, and that they might foster large-scale general public screening initiatives in low-resource settings (Argolo et al., 2020).

The other strength of our study is the performance produced by the machine learning algorithm. Without using any other clinical variable and using only data derived from brief videos of participants, our model elicited an excellent performance (84 % accuracy) in distinguishing ARMS from control individuals. This performance is comparable or even slightly better than other established available risk calculators (Cannon et al., 2016; Carrión et al., 2016; Lee et al., 2020; Oliver et al., 2021; Zhang et al., 2019). Nevertheless, this is still a first proposal that needs to be submitted to larger samples for external validation and real-world implementation, likewise it has been done with all these consolidated calculators.

At last, some of the features used in the model could be traced back to subject's negative symptoms. This is important for some reasons. Negative symptoms are key factors for conversion outcome in the ARMS population (Zhang et al., 2020). However, current assessments for negative symptoms from available interviews are highly ineffective and subjective (Zhang et al., 2022). Thus, providing a tool that could quantify negative symptoms through facial data extracted from short video-recordings is of great potential for clinical use. Additionally, results presented here invite for external validation studies to seek for the underlying biological mechanisms behind negative symptoms and facial expression. Neuroimaging studies, for instance, could investigate the biological ground-truth behind negative symptoms in ARMS states by linking facial movement deficits to specific brain changes.

Our study has several limitations. First, the use of video data alone may also constitute a limitation, as black-box mechanisms might be leading to a hidden bias in the ML model (Loch et al., 2022a). To counter this hypothesis, we analyzed the most used landmarks in the model, and verified that controls displayed an increased variability of data from facial vectors compared to ARMS individuals. This could represent affective blunting and reduced facial expressivity, which is a landmark of psychosis, and has also been observed in ARMS individuals (Gupta et al., 2019). As expected, in our sample negative symptoms were significantly higher in ARMS compared to controls. Ideally, grounding of the current findings on clinical data should be performed with other specific statistical analyses. But we chose in this study to focus on video data alone, to avoid data leakage, and to test the concept of a video-only technology to screen for at-risk mental states at the population-level. Furthermore, the relationship between facial expression and negative symptoms in this sample has already been described elsewhere (Lopes-Rocha et al., 2022). Second, our study has a modest sample size, and the fact that it was conducted in a single center. Findings should be interpreted with caution, as the protocol needs to be replicated in larger samples, as mentioned. Accordingly, it would be advisable to assess if findings of the ML are culture-specific and if the ML algorithm needs to be trained in and to be adapted to different cultures. Third, due to the small sample size, the high performance reached by the model might be result of overfitting, to some extent. To counter for possible effects of overfitting, we adopted the use of Monte Carlo Cross Validation, where the group of 128 participants was divided in 100 different train and test splits, mitigating the effect of the chosen split as we report the average and standard deviation of the metrics obtained on 100 different trained models. However, even though these techniques were adopted, overfitting cannot be ruled out as an important factor in the model. Fourth, due to practical constraints in the conduction of the populational sampling, we were only allowed to interview adults, reason why we could not assess younger individuals and why our mean age is higher than in other studies. Fifth, we did not have any specific instrument to measure depression. Depression is highly comorbid in ARMS individuals and can also cause emotional flattening and altered facial movements, but due to the extension of the study protocol we could not include one more measure besides the ones already included.

## 5 Conclusions

Summarizing, our study generated an accurate ML ensemble algorithm to detect ARMS individuals among a sample of general population participants. This provides an important step toward community detection of ARMS, especially in low-resource settings. Using a brief video paradigm recorded through a mobile phone and providing high-accuracy results in identifying ARMS subjects provides a great public health opportunity to escalate the current findings to larger populations. The extensibility of the current findings should be tested by studying larger and multicultural samples to address external validity, as described in the limitations above. Also, multimodal analysis, using natural language processing and other clinical data should be planned, aiming to ground the present results and to perhaps enhance the accuracy of the algorithm. At last, utilization of the protocol in the follow-up of this cohort should reveal if it is also useful for predicting future mental disorders.

## CRediT authorship contribution statement

AAL and WFG designed the study. AAL, JCA, MTB, LPJ and NMH participated in data collection. JMG, FCA, ACLR, AA and NBM performed data analysis. AAL and JMG wrote the first draft. All authors revised the manuscript and approved its final version.

## Declaration of competing interest

Authors declare they have no conflict of interest.

## Appendix A Supplementary data

Supplementary material 1 Image 1

Supplementary material 2 Image 2
