# Supplementary material for: Detecting at-risk mental states for psychosis (ARMS) using machine learning ensembles and facial features
Source: Schizophr Res. 2023 Aug;258:45–52. doi: 10.1016/j.schres.2023.07.011 (PMC10448183; doi:10.1016/j.schres.2023.07.011)
Supplement: Supplementary file 1 — Supplementary material 1 [file mmc1.docx]

**Supplementary material**

**Supplementary methods**

**Methods S1 — Feature engineering and selection procedures**

**Median Absolute Deviation:**  Information about the behavior of the data can be given by the *median* (med) of the elements. The *median* shows where the data is centered, it is the middle position of the sorted elements, thus, it can be represented as q_n_(0.50)(Dekking et al., 2005). The Median Absolute Deviation (MAD) involves calculating the absolute deviations of the elements from the median and them summarizing these deviations with their median, as depicted by Equation 2:

$MAD={b*M}_{i}(\left| x_{i}-M_{j}(x_{j}) \right|)$, (2)

where *b* is a constant set depending on the distribution of the data, *M_i_* is the median of the absolute difference between each value and the median *M_j_* of the *x_j_* values present on the data(Leys et al., 2013). To ease interpretation, the Median Absolute Deviation will be treated as Variation 1.

**Interquartile Range:** The Interquartile Range (IQR) is a measure of data dispersion that calculates the mid range of the data, serving as an estimation of the variability of the points, in our case, among time(Dekking et al., 2005). To calculate the IQR, first we need to introduce the *pth empirical quantile*: q_n_(p), a number that divides a dataset in two different parts in such way that a proportion *p* of the dataset is less than q_n_(p) and a proportion 1- *p* is greater than q_n_(p), with *n* being the number of elements that the dataset contains(Dekking et al., 2005). Therefore, the IQR is calculated as the difference present in Equation 1:

$IQR=q_{n}(0.75) -q_{n}(0.25)$, (1)

where q_n_(0.25) and q_n_(0.75) being called the *lower quartile* and *upper quartile* respectively. To ease interpretation, the Interquartile Range will be treated as Variation 2.

For each video recording, we used face-alignment(Bulat and Tzimiropoulos, 2017) to extract 68 facial landmarks points from subjects for each frame. Face-alignment estimates facial landmarks with deep learning based methods and returns, at each frame, a 68x3 vector containing the location of each point in the *X*, *Y* and *Z* axes. An example of extracted points can be seen in Figure 1a, where it is shown the facial points used in our final descriptor (circled points).

Videos recorded were checked for any kind of peculiarity that could harm facial landmarks detection: masks being used, glasses that were reflecting light in a way that one could not see the participant’s eyes, interviews being recorded with online video call software (these recordings change the camera being shown according to the person speaking at the moment) or the participant being sideways to the camera. Videos that had these problems were not included on this study

Since some outliers might have been introduced into the predictions from the tool used to extract information (predictions mistakes or some subjects bringing their hands in front of their face during recordings), we decided to mitigate outliers influence in videos by using MAD and IQR on the feature engineering steps, also we decided not to use points 0 to 16 to avoid errors caused by hair movement near to the face jaw. After extracting facial landmark points and ignoring face line points, we proceeded to create the dataset used to train the algorithm, using the 51 points on the *X* and *Y* coordinates and their interactions.

The list of extracted features is displayed as follows:

- *X and Y coordinates:* For both X and Y coordinates, we calculated the interquartile range and median absolute deviation for every point in each frame from the video, eliciting in a total of 204 features. The final name being: “Variation1(P*ii*_*p*)” or “Variation2(P*ii*_*p*)”, with “ii” indicating the face point and “p” the axis.
- *Euler Angles:* We used the faces landmarks to retrieve the Euler Angles, pitch, yaw, roll as depicted on Figure 1b, of the subject’s heads at each frame for every video.  With these values, we stored  the IQR and MAD deviation of each of the angles for each participant’s head, resulting in 6 features named “Variation1(*euler*)” or “Variation2(*euler*)”, with “euler” indicating pitch, yaw or roll. We also calculated the MAD and IQR from the sums of the angles, adding 2 more features that were stored with the names “Variation1(sum_pyr)” and “Variation2(sum_pyr)”.
- *X and Y coordinates outliers:* Even though we decided to use robust statistics that are robust to outliers, when summarizing XY coordinates, we believe that the amount of outliers present for each subject is relevant for classification as they might indicate irregular movimentations. The amount of outliers might be representative to discriminate participants that movimentate more than others, therefore, for each coordinate, we calculated the number of outliers present considering every point detected (a value was considered an outlier if it was higher or lower than the maximum or minimum of a boxplot created with the points), we then divided the number of outliers by the number of frames present for each video, since videos are not always of the same length. This elicited 102 features, named “P*ii*_*p*_irregularPs” with “ii” indicating the face point and “p” the axis. We also divided the total number of outliers present by the total number of frames, naming it “irregularPsPFrame”.
- *Eye Aspect Ratio:* The Eye Aspect Ratio (EAR) is the ratio between the horizontal and vertical lines formed by the eye points detected, as shown in Figure 1c. The EAR was calculated for each eye for every frame on the videos and we use them to store features  in different ways: the left and right EARs, the simple mean between left and right EARs, the product between left and right EARs and the ratio between left and right EARs. After storing each of these values, we calculated the interquartile range and mean average deviation within them. We also calculated how many times the EAR was below the 25% quantile of all EARs measured throughout the videos and divided this number by the number of frames in the video. This elicited 11 features respectively named: Variation1(leftEARs) and Variation2(leftEARs), Variation1(rightEARs) and Variation2(rightEARs), Variation1(ears) and Variation2(ears), Variation1(ears_poly) and Variation2(ears_poly) and Variation1(ears_ratio), Variation2(ears_ratio) and ears_%belowq1.
- *Mouth Aspect Ratio*: Similarly to the EAR, the Mouth Aspect Ratio (MAR), shown in Figure 1d, is obtained by dividing the vertical distances by the horizontal distance of the person’s mouth. We calculated the MAR for each frame and used the interquartile range and mean average deviation within them, named Variation1(mars) and Variation2(mars).
- *Spearman Correlation Coefficient*: The Euler angles calculated are being used to gather discriminative information about how the participants move their heads during the videos. We analyzed that some correlation between points could be as informative as face angles, adding more features to the final classifier, hence we calculated the Spearman Correlation Coefficient between 14 points in the X and Y coordinates: (27, 33), (19, 48), (24, 54), (37, 44), (38, 43), (41, 46), (40, 47), (48, 54), (60, 64). We calculated the correlation between points among the entire video for each video, resulting in 18 features stored as “Corr1_*ii*_*jj*_p”, “ii” being the first point and “jj” the second. We also added the correlation between the MAR and EAR computed in each frame with the name “Corr1_mar_ear”.
- *Angle between points*: We added another angle related feature: the angle between point number 33 and every point extracted from the face, calculating the arctan between these points and then converting the result to degrees. After retrieving the angle between point 33 and all the others for each frame, we calculated the interquartile range and mean average deviation. This procedure produced 100 features, each of them named “Variation1(P33_angle_P*ii*)” or “Variation2(P33_angle_P*ii*)”, with “ii” indicating the face point which the tangent was calculated with.
- *Distance between points*: Additionally to using the angle, we also calculated the distances from point 33 to all the others, using the interquartile range and mean average deviation after calculating this distance for each frame. This procedure produced 100 features, stored in a similar way to the angles names: “Variation1(P33_distance_P*ii*)” or “Variation2(P33_distance_P*ii*)”
- *Partial Autocorrelation Coefficient*: Since we are taking in consideration head movements amongst participants as discriminative information, we added the Partial Autocorrelation Coefficient (PACF) to analyze the behavior of movements frame-by-frame. The coefficient summarizes the relationship of a feature in a time series with the previous observations of this feature. We calculated PACF for every point in the x and y axis with a lag of 1 (the position of the point in the previous frame), resulting in 102 features named P*ii*_*p*_Corr2.

After extraction, the final number of features for each participant was 649. Seeking to extract more information on how these features interact, we performed a combination with repetition of the features from a participant, resulting in a huge 211574 dimension vector, representing the 649 original features added with 210925 features created from repeated combinations of them.

To reduce the size of this final vector, a feature selection step was performed by making use of Gradient Boosting Machines through LightGBM software. We used repeated holdout (with 100 different train and test splits) to train 100 LightGBM algorithms, with the default parameters and fixed random state, and extracted the “split type” feature importance given to each of the 211574 features, adding these values 100 times. After this process, we selected features with summed importance values higher than the 99,95% quantile of all features, resulting in a 116 dimension vector for each participant. Before training our model, we removed features with pairwise spearman correlation higher than 99%, ending with a 100 dimensions vector for each participant. This final vector is composed of “single features” (features not resulting from feature interaction) and “polynomial features” (features resulting from the repeated combination step).

Age, gender and scholar information were added to the final vector before training, scholar information was missing for one of the participants and we imputed the most frequent value depending on the split it is allocated, as explained further.

To ease the interpretation from the features being used, animated figures of participant’s head movement are available at the supplement material (Animated Figure 1 and Animated Figure 2).

**Methods S2 — Metrics**

The F1 Score is the harmonic mean between Precision and Recall (Sensitivity) and is calculated as:

$Precision=\frac{True Positive}{True Positive + False Positive}$,

$Recall=\frac{True Positive}{True Positive + False Negative}$,

$F1=2*\left( \frac{Precision x Recall}{Precision + Recall} \right)$,

it gives equal importance to both Precision and Recall(*Data Mining*, 2012).

Specificity and Sensitivity are metrics used to evaluate how well a model can classify positive and negative outputs. The Sensitivity is an alternative name for Recall, inflicting penalties when False Negative classifications happen, whereas Specificity is calculated as:

$Specificity=\frac{True Negative}{True Negative + False Positive}$,

penalizing models that predict a high number of False Positive outputs(*Data Mining*, 2012).

The Balanced Accuracy is a function that represents the arithmetic mean of the Sensitivity and the Specificity, thus well assessing models trained on imbalanced datasets(Brodersen et al., 2010). The metric is calculated as:

$Balanced Accuracy=\frac{1}{2}\left( \frac{True Positive}{True Positive + False Negative}+\frac{True Negative}{True Negative + False Positive} \right)$.

Receiver Operating Characteristic Curves show the cost benefit of the True Positive Rate (TPR), number of positive samples that are correctly classified, and the False Positive Rate (FPR), number of negative samples that are incorrectly classified. The ROC curve can elucidate the rate of correctly recognizing a positive sample at the cost of making a mistake when predicting a negative sample(*Data Mining*, 2012). The TPR and FPR can also be shown as:

$TPR=\frac{True Positive}{True Positive + False Positive}$,

$FPR=\frac{False Positive}{True Negative + False Positive}=1-Specificity$.

**Supplementary Figures**


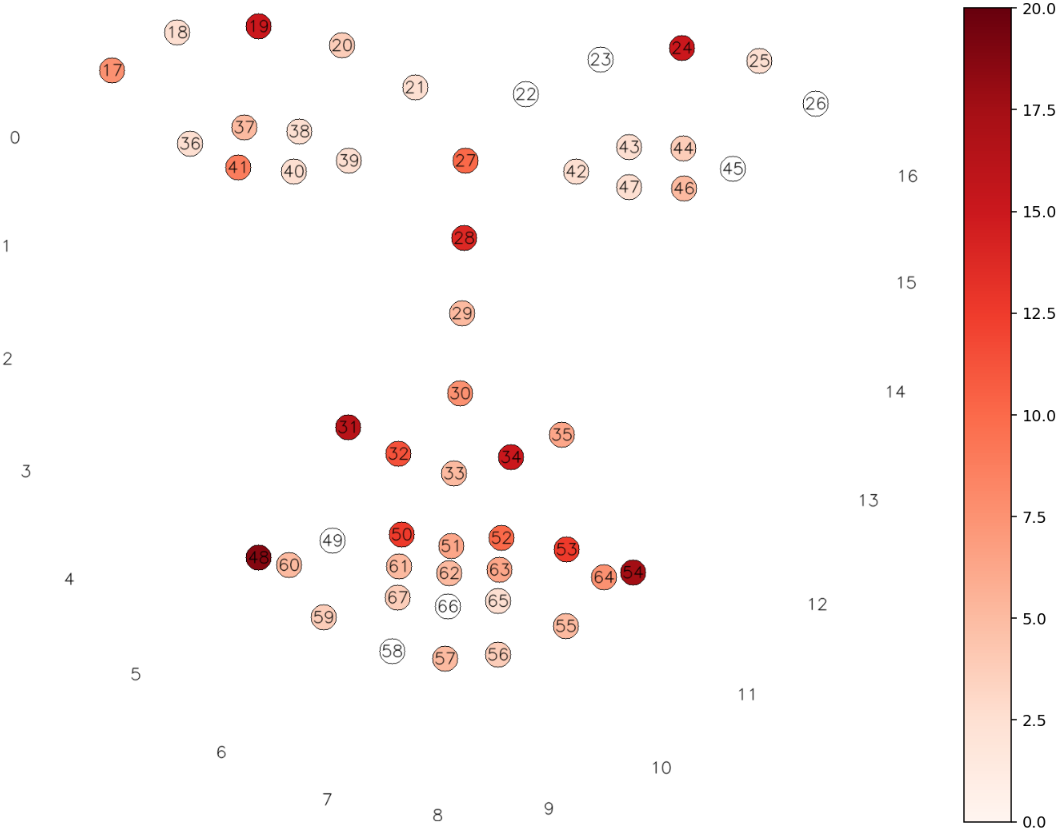


**Figure S1** — Number of times each point appears after the feature interaction and feature selection steps.


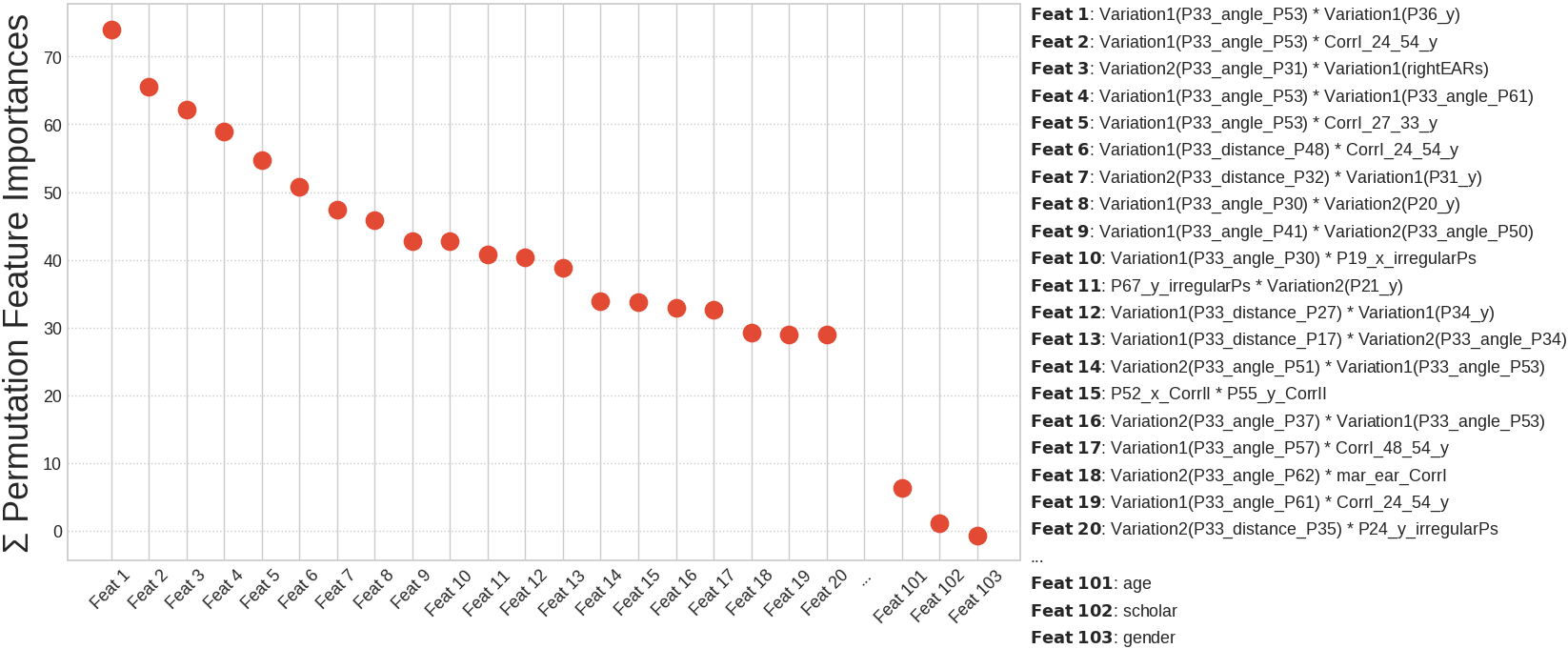


**Figure S2** — Summatory of the permutation importance for each feature in the predictive model after the feature interaction and feature selection steps. The two most used interactions are: Angle between facial landmark 33 and 53 (upper lip) median absolute deviation times facial landmark 36 y axis median absolute deviation (eye corner), and angle from facial landmarks 33 to 53 (upper lip) median absolute deviation times spearman correlation coefficient of facial landmarks 24 (eyebrow) and 54 (corner lip) on the y axis.


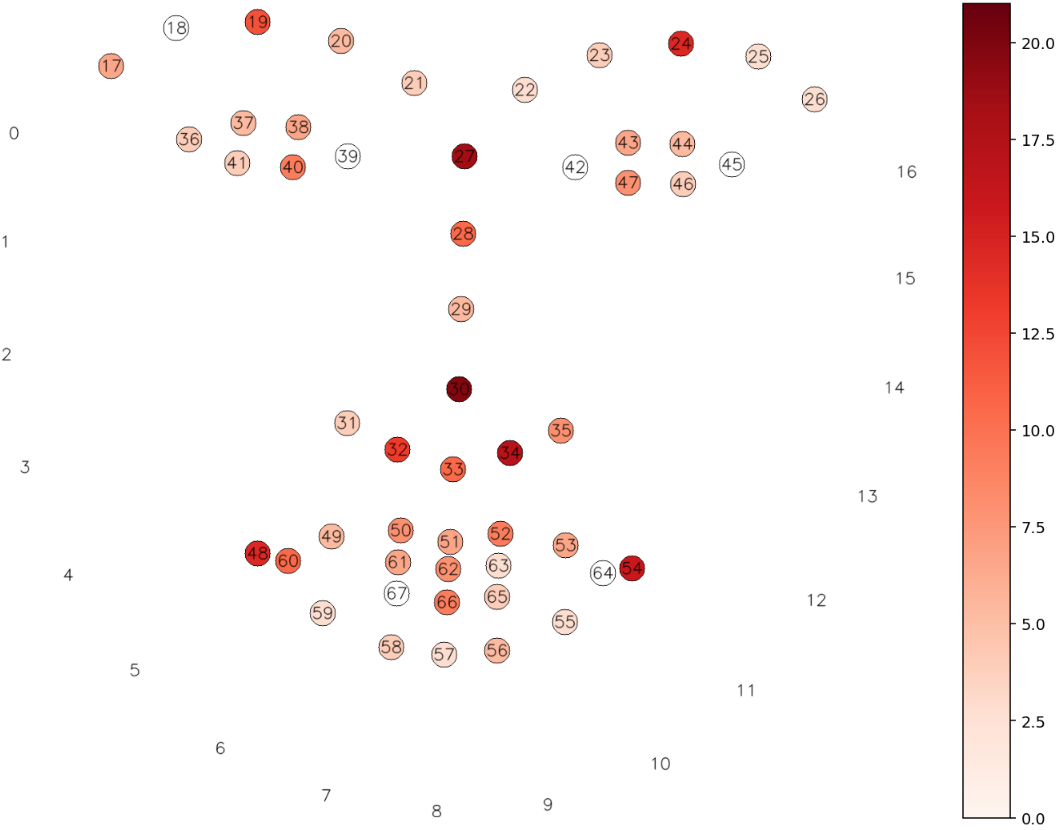


**Figure S3** — Number of times each point appears after the feature interaction and feature selection steps of the MR videos.


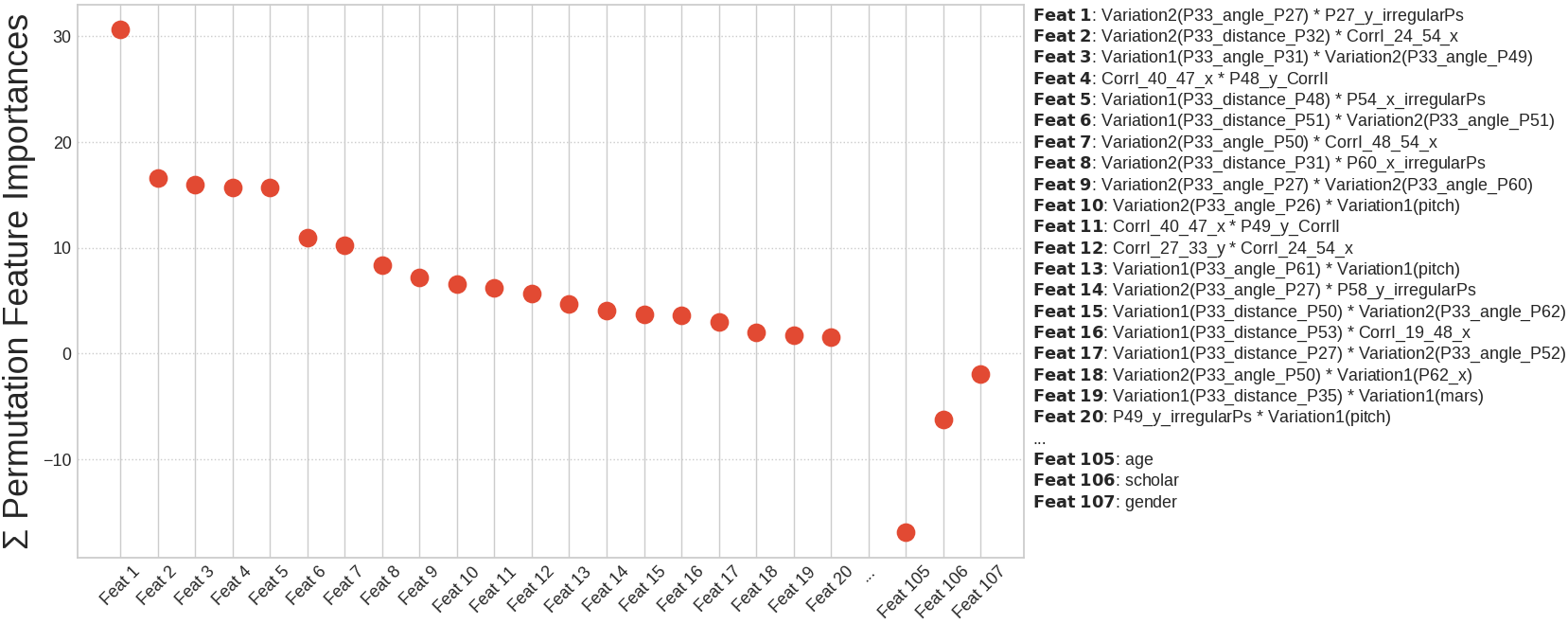


**Figure S4** — Summatory of the permutation importance for each feature in the predictive model after the feature interaction and feature selection steps. The two most used interactions are: Angle between facial landmark 33 and 27 (midpoint between eyes) interquartile range times facial landmark 27 y axis outliers percentage, and distance from facial landmarks 33 to 32 (left nose side) interquartile range times spearman correlation coefficient of facial landmarks 24 (eyebrow) and 54 (corner lip) on the x axis.

| **a)**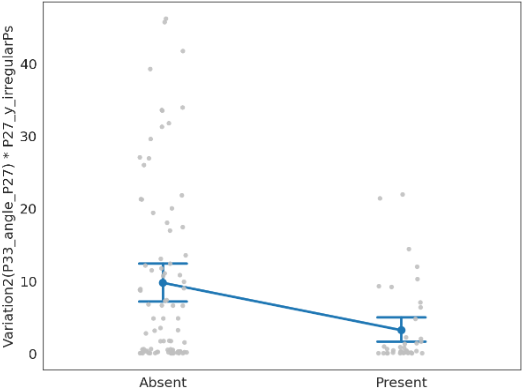 | **b)**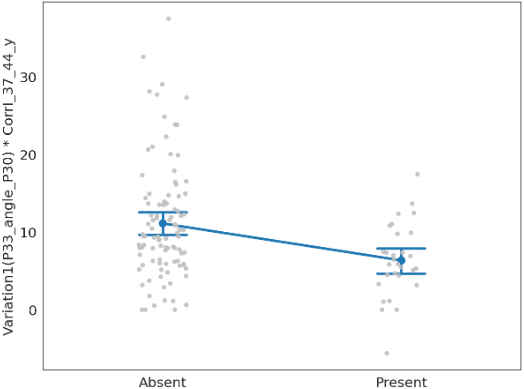 |
| --- | --- |
| **Figure S5:** **a)** Representation of feature “face_33_angle_face27_iqr * face_27_y_outliers” (each point corresponds to one participant) and the estimative tendency of the mean with 95% confidence interval (line joining categories) for the absence or presence from symptom **N1 (Social Anhedonia)**. For this feature, the p-value of the Wilcoxon’s Ranksum test is 0.003. **b)** Representation of feature “face_33_angle_face30_mad * spearman_37_44_y” (each point corresponds to one participant) and the estimative tendency of the mean with 95% confidence interval (line joining categories) for the absence or presence of symptom **N5 (Ideational Richness)**. For this feature, the p-value of the Wilcoxon’s Ranksum test is 0.001. | |

**Supplementary Tables**

**Table S1** — Sample sociodemographic and clinical data of MR videos

|  | **ARMS (n=56)** | **Controls (n=71)** | **p-value** |
| --- | --- | --- | --- |
| Age (mean, SD) | 28.63 ±4.45 | 28.66 ±4.66 | 0.7** |
| Gender (male; n,%) | 18 (32.14%) | 24 (33.80%) | 1*** |
| Years of education 0-9 (freq;%) | 2 (3.57%) | 0 | 0.18*** |
| 10-12 | 20 | 21 |  |
| 13+ | 34 | 50 |  |
| SIPS positive (mean, SD) | 8.73 ±3.04 | 3.80 ±2.37 | **<0.001**** |
| SIPS negative (mean, SD) | 6.05 ±4.50 | 4.41 ±3.57 | **0.004**** |
| SIPS disorganization (mean, SD) | 2.70 ±1.76 | 2.08 ±1.65 | **0.003**** |
| SIPS general (mean, SD) | 5.90 ±3.39 | 3.80 ±3.09 | **<0.001**** |

**Wilcoxon test

***Fisher exact test

**Bold**: significant associations

**Table S2** — Machine learning model performance of MR videos

|  | **Mean** | **Standard Deviation** | **Maximum** | **Minimum** |
| --- | --- | --- | --- | --- |
| F1-Score | 82% | ±7% | 96.97% | 61.11% |
| Specificity | 88% | ±7% | 100% | 68.18% |
| Sensitivity | 81% | ±10% | 100% | 58.82% |
| Balanced Accuracy | 84% | ±6% | 97.06% | 66.44% |
| ROC-AUC | 93% | ±4% | 100% | 79.14% |
