# Supplementary figures and images for: Detecting at-risk mental states for psychosis (ARMS) using machine learning ensembles and facial features
Source: Schizophr Res. 2023 Aug;258:45–52. doi: 10.1016/j.schres.2023.07.011 (PMC10448183; doi:10.1016/j.schres.2023.07.011)

## Slide 1
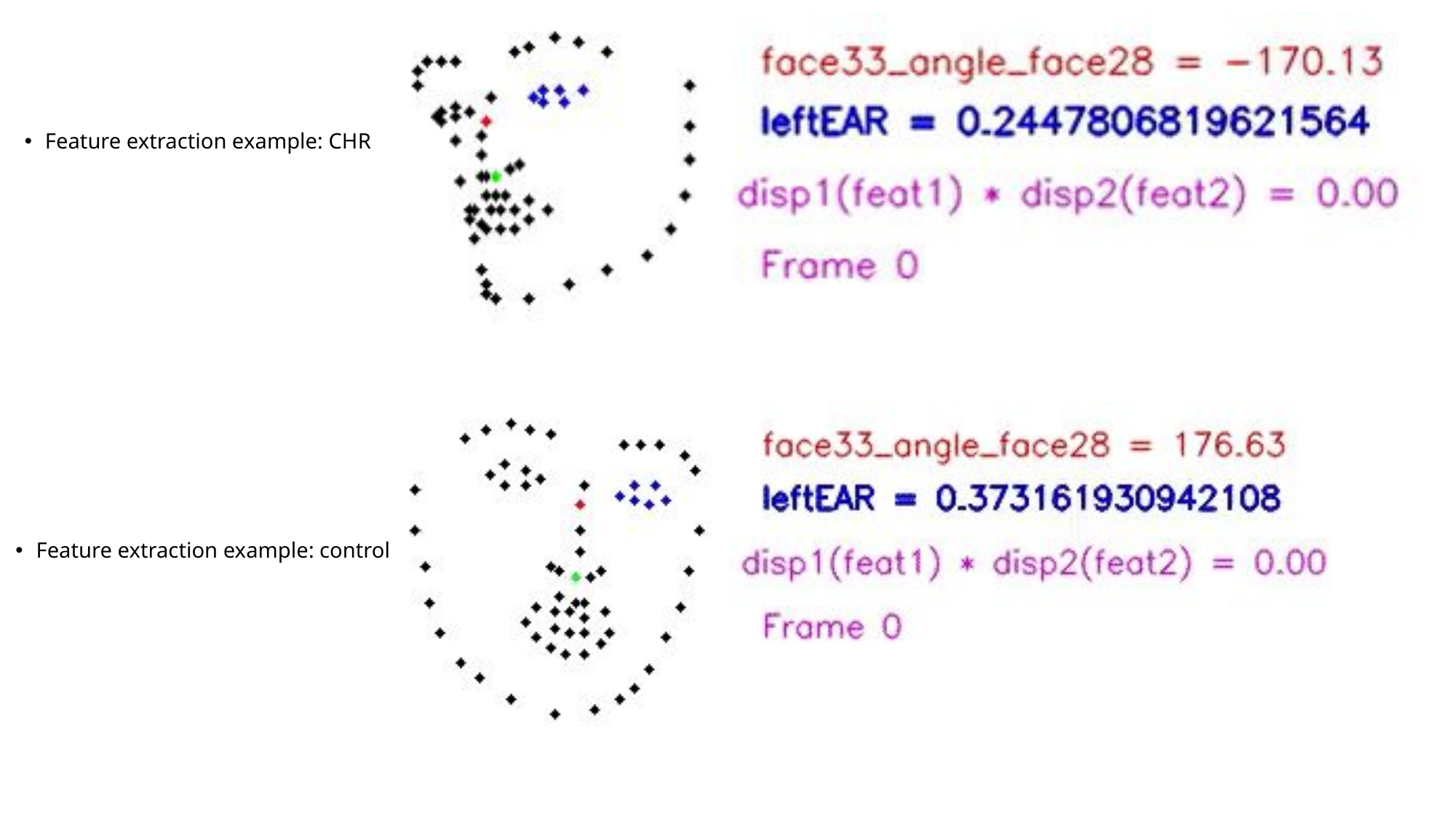

Feature extraction example: CHR
Feature extraction example: control

Supplement: Supplementary file 2 — Supplementary material 2 [file mmc2.pptx]
